# Imaging the Creative Unconscious: Reflexive Neural Responses to Objects in the Visual and Parahippocampal Region Predicts State and Trait Creativity

**DOI:** 10.1038/s41598-017-14729-7

**Published:** 2017-10-31

**Authors:** Morten Friis-Olivarius, Oliver J. Hulme, Martin Skov, Thomas Z. Ramsøy, Hartwig R. Siebner

**Affiliations:** 1 Danish Research Centre for Magnetic Resonance, Copenhagen University Hospital Hvidovre, DK-2650 Copenhagen, Denmark; 2 Center for Decision Neuroscience, Department of Marketing, Copenhagen Business School, DK-2000 Copenhagen, Denmark; 3 Copenhagen Institute of NeuroCreativity, DK-2200 Copenhagen, Denmark; 4 Center for Behavioral Innovation, DK-4300 Holbæk, Denmark; 5 Singularity University, Moffett Field, CA 94035 United States; 6 Department of Neurology, Copenhagen University Hospital Bispebjerg, DK-2400 Copenhagen, Denmark

## Abstract

What does it take to have a creative mind? Theories of creative cognition assert that the quantity of automatic associations places fundamental constraints on the probability of reaching creative solutions. Due to the difficulties inherent in isolating automated associative responses from cognitive control, the neural basis underlying this faculty remains unknown. Here we acquired fMRI data in an incidental-viewing paradigm in which subjects performed an attention-demanding task whilst viewing task-irrelevant objects. By assigning a standard creativity task on the same objects out of the scanner, as well as a battery of psychometric creativity tests, we could assess whether stimulus-bound neural activity was predictive of state or trait variability in creativity. We found that stimulus-bound responses in superior occipital regions were linearly predictive of trial-by-trial variability in creative performance (state-creativity), and that in more creative individuals (trait-creativity) this response was more strongly expressed in entorhinal cortex. Additionally, the mean response to the onset of objects in parahippocampal gyrus was predictive of trait differences in creativity. This work suggests that, creative individuals are endowed with occipital and *medial* temporal reflexes that generate a greater fluency in associative representations, making them more accessible for ideation even when no ideation is explicitly called for.

## Introduction

For over a century it has been argued that the essence of creative thought lies in the process of bringing disparate elements together to form new combinations^1–4^. One central line of reasoning states that the more numerous and remote the associations evoked by a problem, the higher the probability of mediating a new, creative recombination^5^. While creative cognition likely utilizes the full cognitive tool-kit and all its combinatorial powers^6^, associative capacities have long been conceived of as a defining frame for creativity. Indeed, associative capacity is central to theories of creativity and has been related to flatness of associative hierarchy^5^, disinhibition of associative networks^7^, low cortical arousal^8^, and defocused attention^8,9^. Still, whether automatic associative representations underpin the central features of the creative mind has not yet been directly empirically tested. This is likely attributable to the fact that existing creativity paradigms and operationalisations do not provide a viable means of fractionating associative components from other cognitive components involving cognitive control, evaluation, and response selection^6^. In an incidental viewing paradigm designed to circumvent these problems, we set out to test the hypothesis that the neural correlates of sensory-evoked associative responses are predictive of creativity, both reflecting trait-based variability across individuals (trait creativity), as well as variability in creative performance across time (state creativity). We hypothesised that such markers can be elicited reflexively, independently of any creative task, task-set, and their associated confounds.

**Figure 1:**
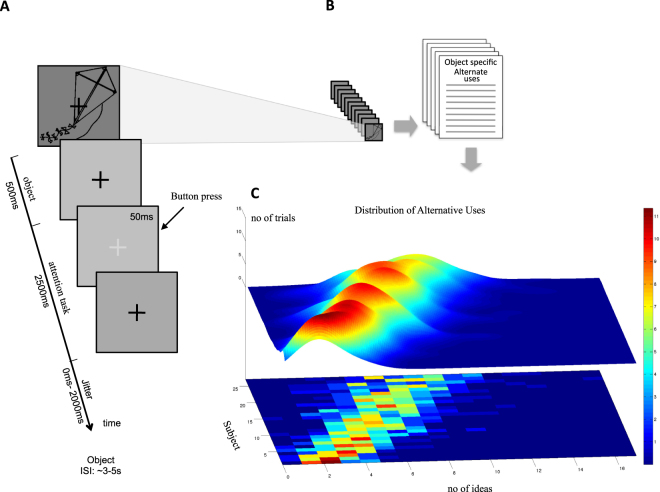
Tasks and performance measures. (**A**) Shows the in-scan task, which consisted of an incidental stream of simple object drawings whilst subjects fixated on the cross and performed a central detection task. The objects were obtained from the CRL-IPNP database (https://crl.ucsd.edu). (**B**) Shows the same images were used in the post-scan creative performance testing, as part of a battery of other creative metrics (not shown). (**C**) Lower, heat map shows the distribution of alternate uses (mean no. of ideas) generated within and between subjects. The subjects are sorted from low to high according to their mean score. The upper surface shows the same distributions as OLS fitted gaussians.

We acquired functional magnetic resonance imaging (fMRI) data in 27 adults engaged in an attentionally demanding luminance-detection task at central fixation (attention task hence, Fig. 1A), adaptively staircased to control attentional load (see Methods). Tracing the neural correlates of associative phenomena is challenging, mainly because associative content cannot be reported in real time without disturbing the automaticity of such associations^10^. Further, since such associations ‘come to mind’ automatically, subjects are most often unaware of their activation. Yet, we know from a plethora of priming studies that such representations are formed, insofar as they reliably bias implicit behaviors^11^, and stimulate intelligible brain activity^12^. To circumvent this problem we designed an incidental viewing paradigm in which subjects were presented with a sequence of simple drawings of common objects, all irrelevant to the in-scanner task performance. Participants were instructed to focus solely on the attention task and did not know why objects were being presented. Immediately after fMRI acquisition, whilst out of the scanner, participants performed a alternate uses test (AUT) for each of the objects that had been incidentally presented (Fig. 1B and D). The AUT^13^ is the most widely used creativity performance test^14,15^ with good test-retest reliability (e.g. refs^16,17^) and shown to be predictive of real-life creative achievements (e.g. ref.^18^) and the effectiveness of creativity training^19^. The incidental design allowed us to assay the influence of automatic encoding of associations evoked by the visual stimuli on *state* creativity, operationalised as trial-by-trial fluctuations in creative performance. Since creativity is multi-faceted, we used a more comprehensive and task-independent composite score here referred to as Creative Potential (CP) to make inferences about individual differences in *trait* creativity (Fig. 1C). Such trait measures are more reliable when based upon multiple tests, showing greater internal consistency and better correlating with real-life creative achievement^20–23^. This trait creativity measure was obtained approximately one year earlier and was based on an array of well-established creativity and creative personality tests, corrected for IQ (Ravens Progressive Matrices, see Methods).

If the number of alternative uses an individual can produce in response to a given object is dependent on the automatic activation of associative representations evoked by the given object, then the neural correlates of those representations, if reproducible over time and generalisable over tasksets, should at least partially predict creative performance with that same object. Further, by the same logic, if more creative individuals have (on average), higher fluency in associative representations, then one would expect that the neural correlates in regions subserving associative memory formation, especially the hippocampus and parahippocampal region^24^, would predict independent measures of trait creativity.

## Results and Discussion

### Behavioral

When debriefing subjects after scanning but before the creative performance task, none reported having paid any specific attention to the incidentally presented objects. All reported being focused on the attention task and when asked, could not remember having seen more than a few objects. The post-scanning AUT where scored by counting the number of uses generated (fluency). Although other performance measures on this test can be computed (originality, flexibility and elaboration), existing data shows that fluency accounts for almost all of the variance in divergent thinking tests^25^. The distribution and analysis of the number of ideas generated across objects and subjects are shown in Fig. S1. On average, subjects generated 5.04 alternative uses per object. We found that subjects demonstrated significant variability in their ability to generate alternative uses across objects (SD = 2.34) (see also Fig. 1C), and that there was significant heterogeneity across subjects’ mean fluency score (mean SD = 1.27). Although we anticipated differences in object performance across time, we found no indication of fatigue effects on fluency performance [*F*(39,1079) = 0.76, *p* = 0.86], and similarly, there was no effect of mean attentional performance (measured as staircase level) on mean fluency scores [*F*(1,26) = 0.45, *p* = 0.51]. For the 40 objects, we found significant variability in object-specific performance across subjects [*F*(1,39) = 2.35, *p* > 0.0001]. A density plot for each object is shown in Figure S1.

### Neural

Using the blood oxygen level dependent (BOLD) signal as an index of regional neural activity, a multiple regression model was constructed to identify brain regions where the BOLD signal response evoked by object onsets was parametrically scaled to the number of alternate uses subsequently recorded in post-scan testing (see Supplementary Methods). Unless otherwise stated, all statistics reported were significant at the cluster-level (p < 0.05 FWE) using a cluster defining threshold of p < 0.001^26^.

**Figure 2:**
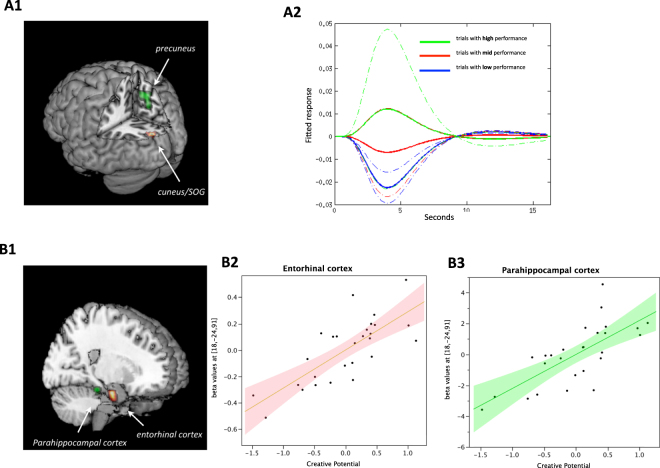
Neural markers of state and trait creativity. (**A1**) Shows object-bound neural responses in the cuneus/superior occiptial gyrus predictive of state creativity, operationalised by performance in the AUT. (**A2**) Shows response profiles within the cuneus and superior occiptial gyrus that scaled parametrically to trialwise variation in AUT score (number of ideas generated per trial). For visualization purposes, trials are split into low, mid and high performance based on a percentile split (33rd, 66th and 100th). Note that these fitted responses are not averages collapsed over trials, they are a means of visualising the parameter estimates in terms of the hemodynamic responses they model. (**B1**) Shows object-bound neural responses predictive of creative potential. (**B2**) Shows the regression coefficients for the entorhinal cortex for the linear effect of trialwise AUT scores and their correlation with CP. (**B3**) Shows the correlation between CP and mean response to object onset in the parahippocampal cortex.

A cluster (*k* = 205) within the left cuneus and superior occipital gyrus (global maximum at x = −18, y = −90, z = 24, *Z* = 3.76) showed an object-bound response that scaled positively and linearly with the number of alternate uses (Fig. 2A). Note that this association between object-bound activity and object-specific variation in creative performance is not trivial. This is because the incidental visual responses occurred automatically in the absence of any conceivable task relevance for the subjects. Whilst it is possible that there are differences in the visual features (e.g. spatial frequency) of the objects that confound subsequent AUT performance, this is unlikely due to the stimuli being simple black line drawings that approximately control for basic features including complexity (see Supplementary Methods). The correlation between left cuneus/superior occipital gyrus and AUT performance thus implies a role for sensory cortices contributing to state creativity.

Object-bound activity in two regions of the right *medial* temporal lobe, namely the entorhinal and parahippocampal cortices, reflected individual differences in trait creativity. Specifically we found that these two regions combined accounted for ~76% of inter-individual variance in CP scores (combined R^2^ = 0.76; see Fig. 2B). Both entorhinal and parahippocampal cortices are implicated in associative memory formation^24^, and have been shown to map onto multivariate sparse encodings of visual associations in an incidental object viewing task similar to that reported here^10^. In the right entorhinal cortex (peak at x = 18; y = −10; z = −24, k = 105, *Z* = 4.15) CP scores were predictive of the linear effect of trialwise variation of AUT scores. In other words, the entorhinal cortex of individuals high in trait creativity showed higher responsiveness to objects that elicited more ideas regarding their use, with this effect accounting for ~58% of the variation in CP score (R^2^ = 0.58, *F*(1,26) = 34.01, *p* < 0.0001). The entorhinal cortex serves as the primary input/output gateway of the hippocampal formation, thus functioning as the main interface to the neocortex^27^. Notably, the entorhinal cortex was broadly activated, both its medial and lateral portions, which would be consistent with it integrating information from both the dorsal and ventral visual streams. The link between creativity and object-bound activity is consistent with the view that the entorhinal cortex connects objects with contextual and associative information^28^. This is also consistent with the idea that objects that triggers stronger entorhinal responses, are doing so by virtue of entraining more associates, that could then be accessible for ideation, cognitive control and evaluation. These results suggest that subjects vary substantially with regards to the degree to which objects automatically trigger associative systems, and that this variability has predictive value for both state creative performance and trait creativity.

In the right parahippocampal cortex, CP scores also predicted the object-bound responses (peak at x = 24, y = −28, z = −18, k = 30, *Z* = 3.81), but here only to the mean object response (averaged across all objects). The higher the CP score the stronger the mean neural response of the parahippocampal cortex to all objects (R^2^ = 0.52, *F*(1,26) = 26.73, *p* < 0.0001). Unlike in entorhinal cortex, this modulation by the CP variate was not significantly related to trialwise AUT performance. This indicates that for individuals high in trait creativity, viewing an object in general triggers a stronger response within the parahippocampal cortex, which can be interpreted as mnemnonic encoding and retrieval^29^, in particularly associations between object elements^24^, and is consistent with recent anatomical evidence showing more regional grey matter volume in the parahippocampal cortex in creative individuals^30^.

We further tested which regions showed object-bound activity that was predictive of individual differences in the mean AUT score, an indication of mean creative performance. We found a positive correlation within the precuneus (peak at x = 8, y = −58, z = 52, *k* = 351, *Z* = 4.17) for mean response to objects (Fig. S2). Of note, the precuneus was part of a set of regions that were generally deactivated during object viewing compared to baseline (Fig. S3). This is consistent with the precuneus being part of the default network, which is predominantly deactivated during cognitive tasks^31^. Hence, the positive correlation with mean performance on AUT (R^2^ = 0.54, *F*(1,26) = 29.35, p < 0.0001) reflects a reduced task induced deactivation (less negative) in high-performing individuals (Fig. 2A). Consistent with these results, individual test scores from the S-A creativity test of divergent thinking has been associated with reduced task-related deactivation in the precuneus during an attention-demanding *n*-back task^32^. The same researchers also found that higher scores in the same S-A creativity test was negatively correlated with regional blood flow at rest in the precuneus^33^. Under the attentional dysregulation hypothesis of creativity (ADHC) (for review see^32^) the lack of deactivation in more creative subjects has been ascribed to an inability to suppress irrelevant information. The most direct evidence for this hypothesis comes from studies of latent inhibition, which refers to the brain’s unconscious capacity to ignore stimuli that experience has shown to be irrelevant. Studies of this phenomenon have found that high levels of creativity are associated with low levels of latent inhibition^34,35^, which in turn has been ascribed to deficits in selective attention^36^. Similarly in tasks measuring mental processing speed, creative individuals have been shown to be slower following attentional intrusions^37,38^. This finding has added credence to the idea that creativity may be related to an attentional dysfunction whereby creative individuals are less able to screen out irrelevant stimuli. This intrusion of irrelevant thoughts may increase the likelihood of getting original ideas^32^. The results presented here merit an alternative perspective that points to differences in retrieval rather than acquisition. First, in the present study, attention levels were individually staircased and parametrically modeled out on a trial-by-trial basis (See Supplementary Material). The ADHC predicts that subjects scoring higher on the alternate uses test, will perform more poorly on the attention task. Yet we found no such relationship (R^2^ = 0.02, *F*(1,26) = 0.45, p = 0.509, 95% C.I [27.6, 35.5]). Furthermore, we found no evidence of a relationship between activity in the precuneus and trialwise performance on the AUT. It therefore seems unlikely that the precuneal activity observed in the present study was related to object-specific processing or that the lack of deactivation was due to attentional deficits.

Given that more information takes additional time to process, the previously reported slowing of responses in creative individuals following attentional intrusion might not be due to additional processing of the externally presented stimuli, but instead to additional processing of a greater internal response from associative memory systems in the parahippocampal region. Consistent with this interpretation, recent evidence has shown that when general information processing speed is taken into account, more creative individuals show superior focused attentional abilities, but become slower when subliminally primed with associated information^20^.

In sum, our results indicate that incidental viewing paradigms provide a fruitful means by which associative capacities can be decoupled from other cognitive faculties for the future study of creativity. We argue that this strategy, in principle provides a viable solution to one of the major obstacles recently propounded to obstruct the neurobiology of creativity^6^. We have illustrated how this approach can be channelled to distinguish between the neurobiological correlates of associative processes underwriting state creativity, from those underwriting trait creativity. Object-specific fluctuations in creative performance were predicted by superior occipital gyrus and the cuneus. Trait creativity however was better predicted by precuneus and *medial* temporal structures, with the precuneus object onset response predicting mean performance in the AUT, the entorhinal response to trialwise fluctuations in AUT score, and finally parahippocampal object-onset response also predicting mean AUT performance. Together these results are suggestive of how the strength of sensory-associative responses might configure the associative components of trait-level creativity. It is important to emphasize that our results are descriptive, and do not allow for causal inference on the link between associative brain responses and creativity. Rather, we propose that the results described here, offer initial support for the hypothesis that the reflexive encoding of sensory inputs in the parahippocampal region, precuneus and occipital cortices are candidate neural markers for the sensorially associative components of creative thought.

## Methods

### Subjects

Twenty-seven subjects (22–32 yrs, mean age 25, 9 males, no exclusions) participated in the study. All were native speakers of Danish and had normal or corrected-to-normal vision and no history of psychological or neurological illness, and had signed a written informed consent form prior to the study. Ethical approval was obtained from the local Committee on Ethics in Biomedical Research “De Videnskabsetiske Komitéer for Region Hovedstaden” (H-4-2012-128**)**, under the Helsinki Declaration. The applied methods were carried out in accordance with the relevant guidelines and regulations. All subjects were naive to the purpose of the experiment but were truthfully informed that they were participating in a study regarding “personality and the ability to detect luminance change”. Participants were recruited randomly from a previous study’s large sample and all who agreed to participate were included. The task-independent psychometric tests presented below were obtained during a separate study conducted approximately one year earlier.

### Non-attended stimuli (covert task)

Forty simple black drawings of common objects were presented centrally on a grey background for 500 ms, with a fixation cross superimposed (Fig. 1A). All objects were carefully chosen from the CRL-IPNP database (Center for Research in Language - International Picture Naming Project, available as freeware at the IPNP website: https://crl.ucsd.edu), so that they comprised as little visual information as possible (i.e. low complexity), yet remained stereotypic and easily identifiable. The ISI was jittered under a uniform probability distribution between 3000 and 5000 ms (100 ms increments). Objects were presented in a randomized order within 5 cycles, so that each object was presented 5 times, yielding a total of 200 object viewings per subject (5400 across all subjects). A single trial (3000–5000 ms) contained (1) an object presented for 500 ms, (2) a period of 2500 ms in which luminance changes could randomly occur, and (3) a 2000 ms jittering period. The temporal frequency of the luminance changes in the fixation cross was approximately half of the temporal frequency of object presentation, but was independently programmed so that more than one luminance change could occur within one object trial. This frequency difference and the nonalignment between luminance changes and object presentations created a task sensation in which the fixation cross was at the center of attention (see below) and objects seemed to *flow* by in the background of attention.

### Attended stimuli (overt task)

Subjects were not informed why the objects were being presented. The task instructions were to concentrate on the fixation cross at all times, and to report via button press each time they could detect a change in fixation cross luminance. Subjects were informed that the subtle luminance changes (50 ms) would occur throughout the experimental task lasting ~15 min. Prior to scanning, subjects were shown exemplar trials inside the scanner, and were given a brief opportunity to get familiar with the task. The fixation cross was visible throughout the entire experiment and was always superimposed over the objects. The subtle luminance changes occurred for 50 ms and were brought about by changing the RGB color from 0-0-0 (Black:+) to a higher value between 100-100-100 (slightly less black:+) and 1-1-1 (no change). An adaptive staircase algorithm (adapted from^39^) actively adjusted the RGB color value for the luminance change in response to performance. The level of luminance change started at 100-100-100 and was modified according to a 2-down, 1-up rule, where the RGB color value decreased one step if the current and previous responses were correct, and increased one step after one incorrect response. The initial staircase step size was set at 10, in order to quickly reach the individuals overall performance level. After 5 reversals (i.e. changes in direction) occurred, the step size was reduced to 5, allowing for a more fine-grained determination of the individual luminance change detection threshold. The purpose of the staircase procedure was not to determine the detection threshold, as the subjects were informed, but to maximize the subject’s attentional load on the fixation cross and to regulate task difficulty based on individual performance. At the same time, this procedure allowed us to use the data from the adaptive staircase to control for individual variations in attention on a trial-by-trial basis in the fMRI analysis. The task lasted for approximately 15 min.

### Psychometric creativity tests

#### Creative Potential

To calculate the creative potential for each participant, we standardized the scores within each of the four tests and averaged across these standardized scores. Each of the psychometric tests making up the Creative Potential measure are described below from 1:4. Note that the alternate uses test, remote associates test, and creative personality scale, were translated into Danish based on the original tests.

1. *Alternate Uses Test* (adapted from^40,41^): Participants were instructed to write down as many unusual uses they could think of for a given common object within 3 min. Participants were instructed not to include ordinary or unrealistic uses for the object. Prior to the tests an example object was provided (paperclip) with allowed and disallowed uses (ordinary use: hold paper together; unusual use: use as an earring; unrealistic use: fly it to the moon). The three common objects used for the test were: Brick, Newspaper, and Pen. We used the traditional measure of trait creativity in this test, which is the average number of uses generated across the three objects (fluency)^42,43^.
2. *Remote Associates Test* ^5^. This test consists of 30 questions, with each question containing 3 words that appear to be unrelated (e.g. Paint, Doll, Cat). The task is to find a fourth word that is associated with each of the three words (i.e. House). The test is scored by adding up the total number of questions solved within 20 min.
3. *Creative Personality Scale* ^44^. This personality measure consists of 30 characteristics that are either typical of a creative personality (e.g. insightful, original, inventive, reflective, unconventional) or atypical (e.g. conservative, cautious, commonplace, conventional). Subjects check off each adjective that fits their personality. In scoring the test, one point is given each time one of the 18 positive adjectives are selected, and one point is subtracted each time one of the 12 negative adjectives are selected.
4. *Openness to experience* ^45^. A global personality trait and part the Five Factor Model (along with extraversion, agreeableness, conscientiousness and emotional stability). It is the personality trait that has been most closely linked to creativity: It has consistently been associated with both trait creativity^46^ and achievement creativity^47^, with higher scores indicating higher creativity. Empirical evidence suggests that openness to experience is associated with a greater capacity to think in original and unique ways, and the tendency to engage in creative activities and interests (for review, see^48^). McCrae^46^ has suggested that although divergent thinking may provide the aptitude for original thinking, openness provides the inclination to actually be creative.

### Post-scanning Alternate Uses Test

Same as the alternate uses test described above, except that there were 40 objects and subjects were given 1.5 minutes to generate ideas per object. Each object was presented for 500 ms on a stimulus computer using the same procedure and objects as in the fMRI session, except that the attention task was omitted. A sound was played when the time was up for each object, and subjects pressed a button to view the next object. To counteract fatigue, subjects were allowed to take a break if needed.

### Intelligence test - Ravens Progressive Matrices

A non-verbal measure of two complementary components of Spearman’s g, often referred to as general intelligence: the capacity to think clearly and make sense of complex data (deductive ability) and the capacity to store and reproduce information (reproductive ability)^49^. This IQ-test is widely used in both research and applied settings due to its independence on language, cultural biases, reading and writing skills. The 60 items in the test become increasingly difficult, requiring greater and greater cognitive capacity to encode and analyze the information. The test is scored by adding up the total number of solved items.

### Scanning parameters

BOLD-sensitive functional images were acquired using a 3-Tesla scanner (VERIO, Siemens) with a 32-channel head coil. Four hundred and thirty contiguous multislice T2*-weighted images were obtained per subject using an echo planar imaging sequence with the following parameters: repetition time (TR): 2150 ms; echo time (TE): 26 ms; flip angle 78**°**. Forty-two sequential, descending 3 mm axial slices were obtained, interleaved per volume, with an in-plane resolution of 3 mm × 3 mm.

### Imaging analysis

Functional data were analyzed using Statistical Parametric Mapping (SPM8). The raw data were reconstructed and morphed into multislice volumes, while being spatially realigned and unwarped to correct for all six motion parameters. Slice-timing correction was applied to correct for temporal differences in acquisition between slices within the same volume, using a Fourier phase shift interpolation, with the middle slice being the reference slice. A mean image was created per volume and spatially normalized to the Montreal Neurological Institute (MNI) template. The images were thereafter smoothed with an 8 mm full-width at half-maximum isotopic Gaussian filter. Condition effects were modeled using the standard hemodynamic response function of SPM8, while low-frequency drifts in the blood oxygen level dependent (BOLD) signal were excluded with a high-pass filter (128 second cutoff). Short-term temporal autocorrelations were modeled using an AR(1) process.

A Volterra expansion of the estimated head motion parameters, as well as aliased Fourier expansions of the cardiac and respiration cycles were included as regressors of no interest in the first-level general linear model (GLM)^50^. The object onset regressors were parametrically modulated by the object-specific number of ideas (i.e. number of alternative uses) generated outside of the scanner. In addition, we included regressors of no interest for the onset of the fixation cross luminance change, error trials (button press with no luminance change and trials with no answer) and a regressor to model out the motor activation related to button presses. Differences in attention levels, operationalised as staircase levels, were modeled as parametric modulators of object onset, using a first- (i.e. linear) and second-order (i.e. quadratic) polynomial expansions.

Group-based statistical inferences were made by entering the individual parameter estimates for the mean response to objects and *first*-order parametric modulation by the object specific AUT score object into separate random-effects analyses. These second-level models were constructed to include CP and mean AUT scores as covariates, respectively, with IQ scores (Ravens Progressive Matrices) entered as a covariate of no interest. We adopted a combination of voxel-level and cluster-level correction to control against false-positives. Given our hypothesis of the specific involvement of hippocampus and parahippocampus in associative formation based on prior research^24,28^, a priori ROI were defined according to templates from the WFU Pick Atlas^51,52^. Results reported from these areas are corrected for multiple comparisons as stated above but within the ROI search volume.

## Electronic supplementary material

Supporting Information
