# Supplementary material for: Imaging the Creative Unconscious: Reflexive Neural Responses to Objects in the Visual and Parahippocampal Region Predicts State and Trait Creativity
Source: Sci Rep. 2017 Oct 31;7:14420. doi: 10.1038/s41598-017-14729-7 (PMC5663854; doi:10.1038/s41598-017-14729-7)
Supplement: Supplementary file 1 — Supporting Information [file 41598_2017_14729_MOESM1_ESM.pdf]

Supporting Information

Imaging the Creative Unconscious: Reflexive Neural Responses to Objects in the Visual and Parahippocampal Region Predicts State and Trait Creativity

Morten Friis-Olivarius[1,2,3], Oliver J. Hulme[1], Martin Skov[1,2], Thomas Z. Ramsøy[2,4,5], Hartwig R. Siebner[1,6]*

[1]*Danish Research Centre for Magnetic Resonance, Copenhagen University Hospital Hvidovre, DK-2650 Copenhagen, Denmark*

[2]*Center for Decision Neuroscience, Department of Marketing, Copenhagen Business School, DK-2000 Copenhage n, Denmark*

[3]*Copenhagen Institute of NeuroCreativity, DK-2200 Copenhagen, Denmark*

[4]*Center for Behavioral Innovation, DK-4300 Holbæk, Denmark*

[5]*Singularity University, Moffett Field, CA 94035, United States*

[6]*Department of Neurology, Copenhagen University Hospital Bispebjerg, DK-2400 Copenhagen, Denmark*

[*] *To whom correspondence should be addressed. E-mail:* Morten@Friis-Olivarius.com

Tabel ST1. Correlation matrix including mean and standard deviation scores. N = 27

Test	Mean	SD	AUT	RAT	CPS	Openness	IQ	Object Ideation Fluency
Alternate Uses Test (AUT)	9.67	3.5	1.00	0.18	0.45*	0.07	0.11	0.58
Remote Associates Test (RAT)	10.67	3.8		1.00	0.08	0.16	0.40	0.24
Creative Personality Scale (CPS)	5.19	3.53			1.00	0.35	0.44	0.50
Openness to Experience (Openness)	131.70	17.33				1.00	0.21	0.31
Ravens Progressive Matrices (IQ)	54.70	3.75					1.00	0.24
Post-scan object AUT test (Object Ideation Fluency)	5.04	1.27						1.00
Creative Potential (CP)	0.00	0.64					0.45*	0.64***

* = $p < 0.05$; *** = $p < 0.001$

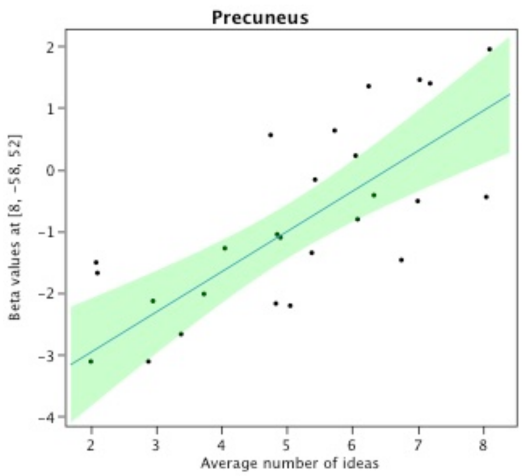

Figure S1. **Mean reflexive response to objects.** For the precuneus the mean neural response to object onset was predictive of mean performance on the AUT. Note that the precuneus is part of a network that was generally deactivated during the tasks, meaning that subjects with a higher mean performance (higher trait creativity) show less deactivation in this area.

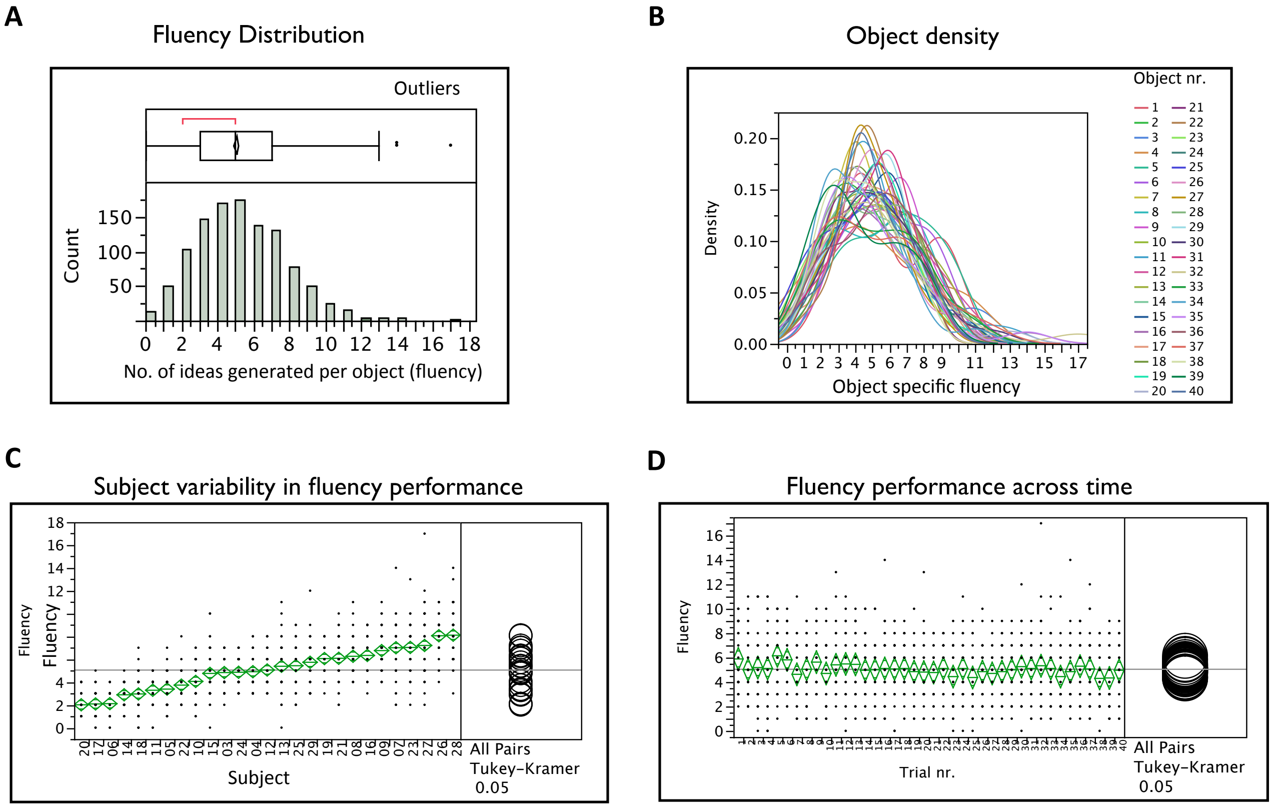

Figure S2. **Behavioral performance on the object AUT test | A)** Normal distribution of the number of ideas generated per object (fluency) across all trials and subjects. Note that the two outliers were considered natural outliers and were not removed from the analysis **B)** Density of the number of ideas generated per object across all subjects. **C)** Individual responses per subject sorted by subject means. An all-pairs Turkey-Kramer analysis is provided to visualize the heterogeneity across subjects. **D)** Number of ideas generated per object across time. The All-pairs Turkey-Kramer is provided to visualize the homogeneity across trials. For both C and D, the mean diamonds represents the confidence interval.

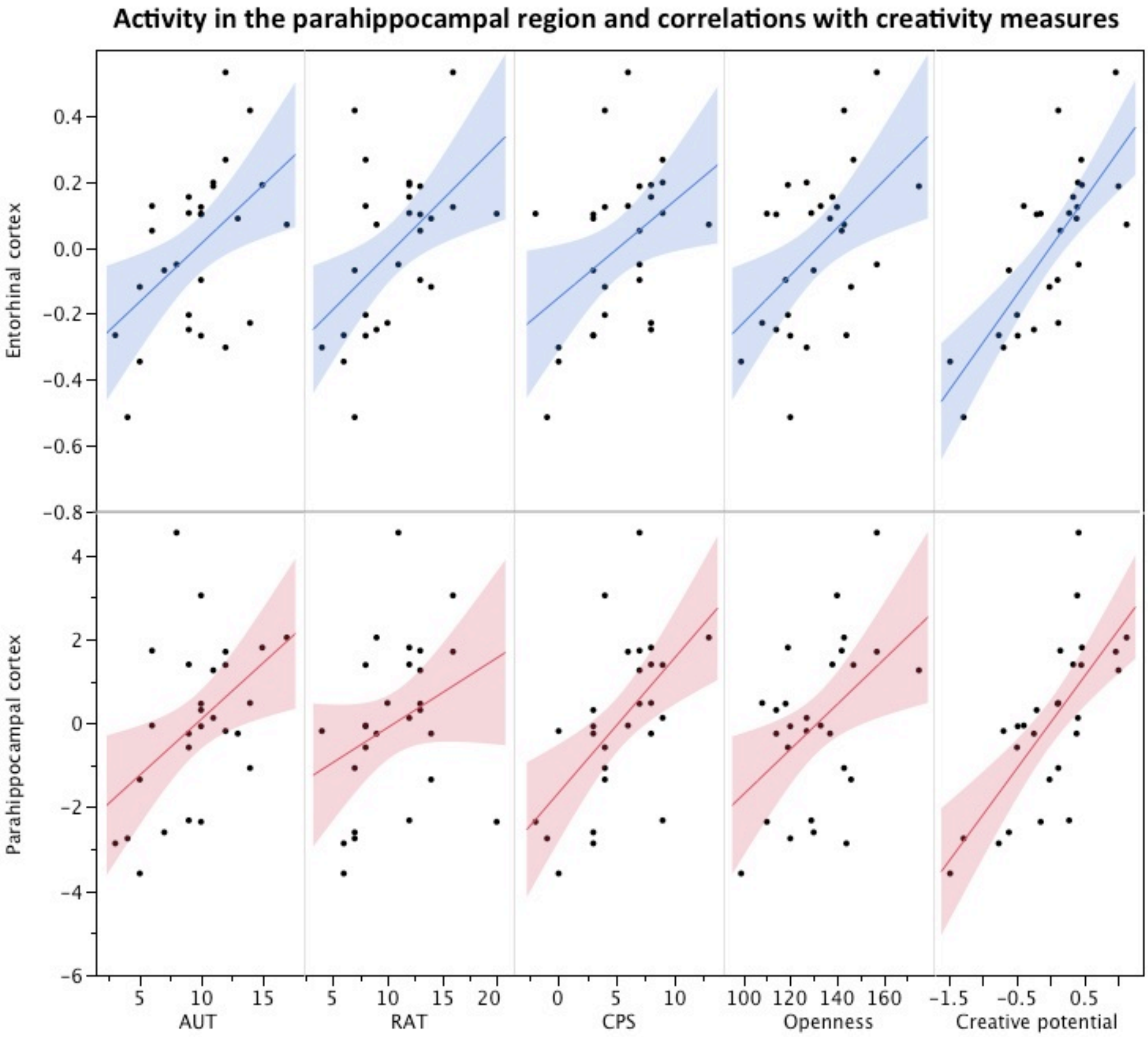

Figure S3. **Parahippocampal Region** | Correlations between the parahippocampal cortex (Betas for the mean object response) or entorhinal cortex (Betas for *1st* order parametric AUT regressor) and each of the psychometric measures of trait creativity, with confidence intervals. Abbreviations: Alternate Uses Test (AUT), Remote Associaites Test (RAT), Creative Personality Scale (CPS), Openness to Experience (Openness).

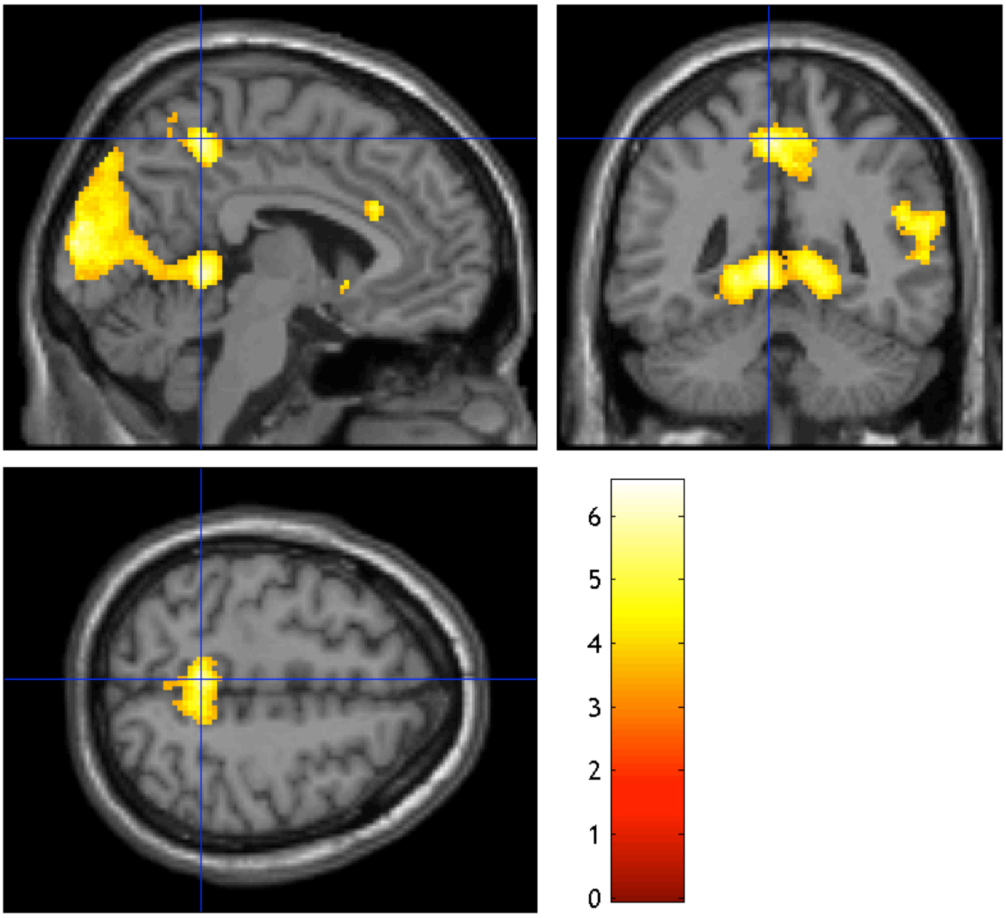

Figure S4. **Deactivations** | Regions that were generally deactivated during object viewing compared to baseline. The blue cross is positioned in the most deactivated voxel within the precuneus and goes through the MNI coordinates; (x = -4, y = -46, z = 54). The color scale shows t values.
